# Editorial and Miscellaneous

**Published:** 1862-03

**Authors:** 


### EDITORIAL AND MISCELLANEOUS.

*Ferrocyanide of Potassium.*—Dr. Newcomer, in the *Lancet & Observer*, after nineteen years careful observation and experience in the use of prussiate of potash is convinced of its great value as a curative agent.

I will now give in few words the class of cases in which this medicine was first used: Invalids, the largest number females of enfeebled habits, relaxed fibre, languid digestion, flatulent, sour stomach, sleepless, and the train of varied symptoms usually attending such a state of the system. Prominent among others is pain, more or less severe, in the stomach and bowels, in the nerves of the face, forehead, etc., but more severe and constant in the head. The bowels may be regular or contrary-wise; pulse often too quick; always irritable, easily excited, and seldom regular for any length of time; hands and feet cold, or inclined to be so, etc., etc. Such cases often gave me much trouble. Anodynes might afford relief for a time, but the distress caused in other respect is often a bar to their use. Tonics would heat the system, dry the tongue, and excite fever; the usual alterative of little or no effect, and a judicious course of diet and general hygienic rules rarely persisted in long enough to secure good results.

After correcting any marked lesion of function of the liver, stomach or bowels that might exist, I would give prus. potassium, ℥ ij., ext. glycyrrhiza, grs. x. to xxx., aqua pura, ℥ ij. Dose, half to a whole teaspoonful, every three to six hours, according to the violence of the symptoms or the promptness of the remedy in relieving them. It may here be said the preparatory treatment did the work, but on this point I made many satisfactory trials, and for years past I give the potash as the first and only medicine used in the case.

Females run down from lactation often present the train of symptoms above described. To many patients in this condition two or four ounces of a solution of prus. potassium would be all the medicine given, and yet speedily cured. Some stomachs reject the glycyrrhiza, and then the formula I use is:

Prus. potassium, ʒ ij. to iv., essence pepperment, gtt. x. to xxx., aqua distillata, ℥ iv. Dose, thirty to sixty drops in elm bark or gum-arabic mucilage, and water. Some take the medicine as it is in a little water.

It is now nineteen years since I began the use of the prus. potassium, and have given it as often as any one preparation of the materia medica, and yet I do not think there was over ten or twelve persons met with who could not take on account of any unpleasant effect on the stomach, bowels or head.

Hundreds are tortured with headache, complaining of no other symptom. Eight out of ten cases of this kind I cure or greatly relieve with this salt, giving in one of the preceding formulas. Neuralgic pains of the face, head or other regions, are often promptly cured by it. In all cases of this nature, the prus. pottassium is depended on. A very intelligent lady, who had for a long time suffered much with headache, said this medicine was essentially a headache medicine, it often giving relief from a severe pain in the head in thirty to sixty minutes, without vomiting, purging, or disturbing effects.

As a stomachic or invigorator of digestion, and hence a tonic, it is in my hands oftener used than all other remedies, and with more success."

In a subsequent part of the article he remarks that he omits his observations on the article as a sedative, considering veratrum viride a more reliable article of the class.

He disclaims the idea of this medicine being a hobby with him, but invites professional examination of the claims he makes for it.

Without giving it a position in any particular class of the Materia Medica, he observes:

"It may be expected that I would class the potassium—assign it place in the materia medica. This I will leave to the next author of such a work. Yet, had I no fear of making this article too long for the editor, much might be said on this head.

I am positive the prussiate of pottasium will reduce the pulse, and is so far sedative. It excites the appetite and promotes digestion,—in some instances in a degree not reached by any other article. I use it in many cases as a tonic. In neuralgia and nervous pains of the head, face, and other parts, in "sun-pain," it will give relief; therefore it is an anodyne.

When successful in asthma, as it has been in my hands, it is antispasmodic or alterative. As a restorer of an exhausted system, from lactation or other causes, it is my most certain remedy. In chest difficulties it has done more than the chlorate of potash for me. In a large majority of neuralgic patients it is my first, my last and only medicine.

If any of our professional brethren adopt the pros. potassiom into the list of their curative agents, I would like at a future period to hear from them, whatever may be their conclusions. I know that men in our profession have their pets or hobby, to which they ascribe virtues that no one else can verify—as Hamilton with purgatives, and a hundred other instances could be found; and I really curious to know how much of all the varied qualities assigned the prus. pottasium in this article will be found by others. No one could be more careful than myself, and nineteen years seems to be time enough to establish a truth in therapeutics."

---

*Cut and Thrust Wounds in the Intestinal Canal.*—Dr. B. Weber, in the same journal, after observation and large experience from 1812, says: "All hitherto recommended methods of operation and manipulation to heal wounds of the intestines can not only be entirely dispensed with, but since they do not further the object of healing, they are rather impeding it, and consequently objectionable."

He asserts that unless the intestine be entirely severed all wounds are immediately closed by its muscular fibres. The object of treatment is to prolong the stage of simple exudation of coagulable lymph until its organization, a period seldom beyond eight or ten days. He directs, therefore, closure of the external wound by sutures, if necessary, to prevent escape of the intestines, and then assiduous application of cold, by cloths frequently wrung out of ice water, by bladders of pounded ice or snow, or, in the absence of these, Schmucker's or other freezing mixtures.

Venesection in plethoric habits. Enemata of mild character, to secure evacuation of the bowels, and generally the antiphlogistic regimen. He observes:

"If the intestinal canal is entirely severed transversely, if necessary, enlarge the external abdominal wound, and try to bring the eed of the intestinal canal attached to the stomach into the intestinal wound, where it can be fastened by a loop fastened in the mesenteries. The pressure of the intestines generally favors its remaining in place. It is to be particularly taken care that the external serous membrane of the intestinal canal be brought in contact with the edges of the wound of the abdominal integuments, since the inner mucous membrane is unfit for exudation of plastic lymph. By resorting to the above mode of treatment, the worst that can happen is an artificial anus, since by application of a loop to the mesentery the edge of the lower end of the intestine by attraction is brought in contact with the lumen of the upper end, and sometimes a perfect union of both ends is accomplished, and the alvine evacuation which for a period took place unnaturally through the abdominal wound, is going on in a natural way, and the abdominal wound finally will close entirely.

For the latter case I can bring proof in a person treated by me many years ago, in Germany, who is now living in Cincinnati."

---

*Test for Diabetic Sugar.*—Dr. Wm. Roberts, of the Manchester Royal Infirmary, publishes in the Edinburgh Medical Journal a simple clinical method of estimating the amount of sugar in diabetic urine. It is exceedingly plain and satisfactory. The method will be appreciated at once by a quotation of brief extent:

"The only instrument needed is the urinometer (with the use of which every practitioner is familiar) and two phials, one of twelve ounces, and the other of four. The principle of the test is most simple. *It is to take the specific gravity of the urine before and after fermentation, and from the loss of density occasioned by the conversion of sugar into carbonic acid and alcohol, to calculate the amount of sugar destroyed.*" A lump of German yeast of the size of a small walnut is added to say four ounces of urine in a twelve ounce phial. This is loosely corked or covered with a slip of glass and allowed to stand twenty-four hours, when the process under the ordinary temperature of living apartments will be complete.

A similar quantity of the urine which has had no yeast added to it, may be kept side by side, and then the density of the two observed. "The difference between the two densities is thus ascertained, and every degree of density lost, indicates one grain per fluid ounce of sugar in the urine."

The practical details are obvious.

---

*Holloway's Pills.*—The formula for this much be-advertised nostrum is, ℞—Aloes grs. lxii; Pulv. Rhei, gr. xxvj; Piperis, gr. viij; Croci, gr. iij; Sodae Sulphat. gr. iij. Ft. massa in pil. cxliv divid.

---

*American Surgery Abroad.*—The American Medical Monthly welcomes the return of Dr. Sims from Europe.

"He has had," says the Monthly, "an opportunity, and a deserved one, of establishing in the eyes of scientific men in Europe his right to be considered the originator of a mode of operation which, even in the brief space of time since he first promulgated it, has been of immense benefit to suffering humanity, and of which the most persistent and unwarrantable efforts have been made to deprive him. His reception in Paris was most gratifying—not only was he called upon to operate before crowded classes in five of the largest hospitals of that city, but the most eminent surgeons confided their private patients to his professional skill. Fourteen cases, only four of which were not *unusually bad*, and fourteen cures! Such is the brief record of his labors in the Capital of the Medical World. We doubt if a similar score had ever been run up there before. 'I was certainly very lucky,' says the doctor modestly. The one of us who has had the most experience, will probably be best able to estimate the 'luck' at its true value. The *Times*—not the English Blood and Thunder War-sheet, but its more peaceably-inclined namesake on this side the water—recently contained a letter from its special correspondent in Paris, which was almost entirely devoted to the consideration of Dr. Sims' visit and success. He mentions a case which possesses considerable interest in view of the question of the employment of anæsthetics, which has recently been brought so prominently before the profession in this city.

"It was that of a young Countess, whose case had been pronounced hopeless by the leading surgeons, and who was placed under his care by Nelaton. A feature of the operation was her narrow escape from death by chloroform. The method adopted for the restoration was both novel and rational, and we are told that it is now generally followed in similar cases in that city. It consisted in "throwing the head of the patient down, and the feet in the air at an angle of abont forty-five degrees. By this manœuvre, the brain and heart, with an increased quantity, receive an increased amount of stimulus, from the blood, and life is preserved until the volatile poison has spent its force, and is dissipated.' The ordinary measures, friction, artificial respiration, and evulsion of the tongue, were also practiced."

---

*Dysentery.*—Dr. Aug. Baudon, in the Bulletin General de Therapeutique, chronicles successful treatment of "a dozen very severe cases of dysentery, characterized by tenesmus, prolapsus ani, bloody stools, 20, 30 to 40 a day, and extreme prostration by the exhibition of the solution of perchloride of iron." He employed also two or three warm water enemata daily, containing from 12 to 25 drops of the solution, with the addition of laudanum if pain was excessive. Duration of disease from four to eight days.

---

*Indian Remedy for Small Pox.*—Herbert Miles, M. D., a Surgeon in the British army, states that the Indians in Nova Scotia employ with very marked advantage an infusion of *Sarracenia Purpurea* (pitcher plant). The effect of a large wineglassful is to bring out the eruption freely. "After a second and third dose, given at intervals of from four to six hours, the pustules subside, apparently loosing their vitality. The patient feels better, at the end of each dose, and in the graphic expression of the *micmac*, knows there is a great change within him at once. In a subject already covered with the eruption of small-pox in the early stage, a dose or two will dissipate the pustules and subdue the febrile symptoms. The urine, from being scanty and high-colored, becomes pale and abundant, whilst from the first dose the feelings of the patient assure him that the medicine is killing the disease."

The constitutional symptoms subside in three or four days, although, as a precautionary measure, patients are kept quiet until the ninth day. It is alleged that no pitting or discoloration follows. The Indians regard it moreover as a preventive and antidote, and drink the weak infusion during the prevalence of the disease.

---

*Irritable Bladder.*—An infusion (ounce to pint) of Triticum Repens is extolled in irritable conditions of the bladder, whether from inflammation, gonorrhœa, calculus, or obscure diseases. It is asserted to be much preferable to buchu. The plant should be gathered in the Spring, shortly before the leaves appear. Improvement, if produced at all, will be immediate. If none can be observed in four or five days, it is useless to continue the remedy.

---

*Otalgia.*—The *Gazette Medicale de Lyon* recommends in severe earache, when other remedies have failed, equal parts of chloroform and laudanum introduced to the meatus on a piece of cotton. "The first effect produced is a sensation of cold, then there is a numbness, followed by scarcely perceptible pain and refreshing sleep." This is not novel, but good.

---

*Researches on the Changes undergone by Cane Sugar in the Intestinal Canal.*—Dr. Kœbner has investigated this subject, and the following are his conclusions :—1. Cane sugar undergoes no change when left from two to four days, at 104° Fah., in natural gastric juice obtained from a dog. 2. Neither is cane transformed into grape sugar in the living stomach. 3. Lactic acid may, after a time, be detected in a mixture of cane sugar, by means of bile which has been freed from mucus. 4. In the ileum cane is transformed into fruit sugar, at least the presence of the latter can be detected in the ileum by means of the polarizing apparatus. 5. Sugar is largely absorbed by the stomach, duodenum, and small intestines. 6. Cane sugar is partly transformed in the latter into lactic acid and fruit sugar. 7. In dogs and rabbits cane sugar occasionally passes unchanged from the intestines into the portal blood, and this probably is the reason why dogs fed on sugar excrete a smaller quantity of urea. 8. Cane sugar, even when eaten in quantity (by dogs), does not reappear in the urine: but after a saccharine diet, the amount of uric acid appears to be increased. —*N. Syd. Soc. Year Book*, 1860.

---

*On the Formation of Sugar and Amyloid Substance in the Animal Economy.*—Dr. R. McDonnell puts the question—Is the liver endowed with the power of converting amyloid substance into sugar during life, and health? In order to elucidate this question, he withdrew, by means of a catheter, blood from the right side of the heart in the living animal, and the results were—

1st. In twelve experiments made on dogs, for some weeks before fed exclusively on meat, traces of sugar were found in the blood of five; there was no sugar found in the blood of the remaining seven.

2d. In four rabbits fed on boiled eggs, meat and butter, for some days, no sugar was detected in the blood drawn from the right side of the heart.

3d. In three dogs, fed on mixed diet, and three rabbits, fed on carrots, potatoes, etc., sugar was found in the blood of the right side of the heart, and in equal quantity in blood from the carotid.

4th. In three rabbits, fed on vegetables, sugar was found in the blood withdrawn during life from the right side of the heart; but double, and in one instance more than treble, the amount was in the blood removed from the same locality after the animals were killed.

Hence, says McDonnell, one seems in some degree justified in concluding that in vegetable-eating animals the blood is normally saccharine; but that the liver does not form and pour out into the blood of the hepatic vessels sugar specially derived from the transformation of the amyloid substance.—*Ibid.*, from *Dublin Hosp. Gaz.*, May 15.

---

*Chorea Dependent on Dental Irritation.*—Dr. J. J. Levick, in *Am. Jour. Med. Sci.*, recalls attention to the frequent dependence of chorea upon the irritation of dentition or of carious teeth. Many cases appearing about the period of second dentition may be relieved by lancing the gums or drawing the first teeth. The same rule holds with reference to carious teeth.

*Sulphuret of Lime* is recommended by Dr. LAVAN, in *Championner's Journal*, to promote the regeneration of the substance of bone. He was led to its use by observing the enlargement of the joints of the fingers of those who used this agent diluted in olive oil in frictions for scabies. He has for many years prescribed frictions with sulphuret of lime on the heads of ricketty children where the fontanelles were excessively large or remained open abnormally. He deems it similarly efficacious in facilitating reproduction of bone in fractures, exsections, &c. In rickets he found it to act with "surprising rapidity."

---

*Chlorate of Potash again.*—Dr. S. H. SMITH, of New York, in the *Am. Med. Times*, believes that the greatest value of the Chlorate of Potash is as a prophylactic. Given with *Quinine and aperients*, he has seen it repeatedly stave off attacks of epidemic fevers. So also it is "a big thing" in that state of constitution favoring boils, felons, whitlows and carbuncles. He, moreover, conceives it to have acted miraculously in certain mental diseases, associated with an unusual lividity and coldness of the lips, extremities, and sometimes tip of the nose, showing an embarrassed capillary circulation, perhaps ascribable to some morbid condition of the circulating fluid itself.

It is evident that Dr. Smith believes that Chlorate of Potash is a power in the medical Israel. He will recover.

---

POISONING BY IODIDE OF POTASSIUM.—A man, thirty-eight years old, who had been suffering from sciatic rheumatism for one year and a half, but who was in other respects healthy, took from an unprofessional person a mixture consisting of two drachms of iodide of potassium, four ounces of water, and one ounce of syrup, in doses of one tablespoonful every half-hour. In the whole thirty-six grains of the iodide were taken. After the second dose symptoms of poisoning commenced and continued to increase during three hours, when the patient presented the following conditions. His head was hot, his face puffed, his eyes swollen, sunken, and very sensitive to light.

The submaxillary region was swollen, there was great restlessness, singing in the head, sharp pains in the head, and great exudation of mucus and saliva from the mouth; there was no particular smell perceptible from the mouth; the inclination was for sickness, but there was no pain in the lower portion of the body. In the circulatory system there was nothing abnormal. As a drink, starch flour was ordered in water, vomiting was induced by the process of tickling the fauces, leeches were applied to the temples and to the back of the ears, and cold cloths were put to the head. In twelve hours the pains ceased, and in forty-eight hours the flow of saliva, the symptoms of swelling, and the other signs of poisoning had vanished.

Clarus, who narrates this case from the 'Nass. Med. Jahrb., p. 747.' 1861, where it is recorded by Dr. Orth, remarks upon it that it is one of interest, because the symptoms called forth by the iodine were pure, and were not interfered with by the symptoms arising from any other disease; because no general symptom of disease was exhibited except nausea; and lately, because the symptoms induced followed so small a dose as thirty-six grains of the salt, in a strong, young, and not oversensitive man.—*Schmidt's Jahrbucher*, Band, iii., Jahrgang 1861.—*Drug. Circular.*

---

*Quinine and Veratria in Typhoid Fever.*—VOGT, on comparison of tabulated cases, prefers veratria to quina in the treatment of typhoid fever. The medical mutiplication table was seemingly never more improperly invoked, as the two articles have nothing in common, and are adapted to almost totally different cases. Of three cases of typhoid fever, side by side, one might be benefitted by the veratria, the next by quina, and the third seriously injured by either. Such observations as those of Dr. Vogt's must injure the profession in the view of truly scientific thinkers.

---

*The Letheon Patent.*—Dr. W. T. G. Morton having instituted a suit under his patent for the use of Ether as an anæsthetic, against the N. Y. Eye Infirmery, the presiding Judge (Shipman), after hearing considerable testimony, having come to the conclusion that the patent was void, and moreover the subject matter was not patentable, directed the jury to find a verdict for the defendants.

Where will the indefatigable W. T. G. M. "turn up" next? Nevertheless, in justice to Boston, if not to W. T. G. M., it must be admitted that anæsthesia, as a practical thing, was born in that city—W. T. G. M. being accoucheur in chief, Prof. Jackson counsel (or rather accessory after the fact), and our venerable confrere, the *Bost. Med. Journal*, the first to chronicle the event.

---

*Homœopathy in the Army.*—The subjoined terse resolutions were prepared by Dr. Valentine Mott, and at his suggestion adopted by the Academy, although "the Napoleon of American Surgeons" was not himself present, in consequence of an unlucky fracture of a metacarpal bone, which latter we earnestly trust will not permanently interfere with the surgical ambidexterity of the professional veteran.

"*Whereas:* Petitions have lately been presented to the Senate and House of Representatives of the United States, for the employment of homœopathists as surgeons in the Army; therefore,

*Resolved*, That the New York Academy of Medicine deem it their duty in the interest of the Army, respectfully to protest against the employment of such practitioners, for the following reasons:—

"1st. That the practice wherever subjected to accurate observation has failed to establish itself in any hospital.

"2d. That in the countries where it originated and attained its fullest degrees of development, it has not been introduced into the army or navy.

"3d. That it is no more worthy of such introduction than other kindred methods of practice as closely allied to quackery.

"4th. That such appointments would dissatisfy and dishearten the Medical Staff of the Army, who understand the true character of homœopathy, and who have entered the service of their country, with confidence that the government would strive to elevate the standard and promote the efficiency of the Medical Staff—results surely to be defeated by the appointment of homœopathists.

"*Resolved*, That a copy of the above resolutions be sent to the Hon. Ira Harris of the U. S. Senate, and the Hon. F. A. Conkling of the House of Representatives, with a request that the resolutions be presented to the two houses of Congress."

---

*The Citro-Ammoniacal Pyro-Phosphate of Iron.*—Prof. E. N. Chapman, of Brooklyn, in an elaborate article in the *Bost. Med. Journal* recommends the (briefly) Pyro-Phosphate of Iron as tasteless, elegant in appearance, and unlikely to disagree with the most delicate stomach. In addition to the full powers of the chalybeates, it affords new virtues giving "this special compound advantages possessed by none other in the Materia Medica. This arises from the pyro-phosphoric-acid." The large physiological importance of phosphorus as an element especially of the great ganglionic centres; the part it plays in cytogenesis; its general presence in all the solids and fluids of the body; its thereapeutic powers as a cerebral stimulant and aphrodisiac—are severally alluded to, and defect in its reception or assimilation considered as morbific.

Prof. Chapman thinks the presence of Phosphoric acid and elemental phosphorus communicates especial value to cod liver oil; that the combination, as in many other cases, gives an article possessed of new and superior powers.

But, he observes, the liberation of phosphorus in the oil by the processes of life will be slight compared with that set free in the blood by pyrophosphate of iron; since the iron is immediately assimilated and appropriated in the processes of nutrition by the red globules of the blood.

"Hence we discover the reason why the oil will augment the deposition of the fat, and, when oxidized, will augment the activity of all the various functions, and why the stimulation from this oil is far less than that from the pyrophosphate of iron. Thus these two medicines afford a means of introducing phosphorus and its acid into the system, a point otherwise difficult to be attained; and secure certain peculiar medicinal results through the nature of their combinations.

Practical, clinical facts, the only reliable foundation for medical practice, confirm, in my experience, the views thus presented on a therapeutical and physiological basis.

We have employed the citro-ammoniacal pyrophosphate of iron, in certain conditions, with the most marked and gratifying results.

Whenever the blood becomes thin and watery, there are, almost invariably, troublesome attendant symptoms, seriously retarding the restoration of the patient to health. In all, there will be a lack of nerve-power, from the hydræmic state of the circulation. Hence, could we, temporarily, augment the stimulating properties of the blood, whilst we are administering the iron, we should prepare the way and present the conditions required for its assimilation, which otherwise might be impossible. Experience has taught most physicians this practical fact, and the indications have usually been fulfilled by the simultaneous use of wine and iron. We have found the pyrophosphate singularly appropriate under these circumstances, and as superior as a natural excitant must ever be over any substitute we may devise. Persons who have been over-worked by mental application and prostrated by disquietude and care, or persons who have a shattered nerve-power from some constant source of bodily suffering, have a thousand anomalous symptoms dependent on an imperfectly generated and distributed nerve-power—such as wakefulness, trembling spasmodic movements, palpitations, &c. For this class of symptoms, the pyrophosphate of iron often affords relief in two or three days; and thus prepares the way for the ultimate cure that may be expected from the martial salts. Many times, patients have expressed wonder at the calming and tranquillizing effects of the medicine; not only in mere functional aberrations and irregularities, but also in cases where actual disease existed in the nerve-centres. In both instances, the stimulation is immediate and transient, and can be of no avail, except by removing irregular nervous distribution; whilst the iron is appropriated more readily by the organic forces now freed from a great source of disorder.

After detailing several cases illustrative of the therapeutic power of the agent he adds:

"For all the varied and anomalous symptoms of hysterical patients, which are usually some phase of irregular distribution of the nervous influence, the pyro-phosphate acts with singular efficiency; diffusing and equalizing the nerve-power, and thus secondarily restoring a more active capillary circulation and a more healthful play of all the functions. Cases illustrative of this point are unnecessary in the milder forms of nervous disease, since the claims of our remedy are sufficiently vindicated in the severer ones hitherto mentioned.

The pyrophosphate of iron has another property scarcely to be expected, and one we should never discover except by actual observation. All of the common preparations of iron are apt to oppress the stomach, coat the tongue and destroy the appetite, especially when the patient is much debilitated. Many, from a delicate, sensitive organization, cannot, under any circumstances, take iron with profit, it being, in their language, too heating. The pyrophosphate is friendly to the stomach, will never cause any irritation of the gastric surfaces, and, to our knowledge, has never disagreed with any patient, however incompatible the other forms may have been. Besides, it appears to possess a tonic power, and will restore the appetite and digestion after the failure of bitters, quinine, wine, &c., often in extreme cases of anæmia, amenorrhœa and chlorosis, as we have witnessed in many instances in our obstetric clinique. It seemed to afford just the grade of stimulus required by the stomach, and the improvement, thus initiated, continued without interruption, under this single remedy, to the complete cure of the patient. This acceptability, friendliness, corrigent and roborant action of this form of iron on the digestive organs is a valuable peculiarity which renders it, in many states of disease, superior to all others, and perhaps to any drug whatsoever. Besides, its tastelessness, when dissolved in syrup, is a great recommendation in this age of sugar, when patients desire to die *sweetly*, and will not endure anything nauseous or unpleasant, though death be knocking at the door. This we might expect in children who *bring* up their parents to a tolerably high state of discipline, and issue their orders of command from the cabinet councils of the nursery. We, medical men, taking the world as we find it, are obliged to render our doses as palatable as possible for babies, both great and small. This object, without detriment in the choice of our means, is singularly and notably attained by the use of the syrup of the pyrophosphate of iron."

---

*Religious Excitement.*—The Irish Lunatic Asylum at Ulster chronicles a large increase of accessions consequent upon two months of general protracted religious excitement. It is assigned as the cause of insanity in 97 males and 86 females.

*Prof. Dixi Crosby, of New Hampshire*, has been nominated to the highly important office of Railroad Commissioner for that State. This is a compliment of a high order to a most estimable and deserving man, more peculiarly grateful as the nomination was given by political opponents as well as friends, Dr. Crosby not belonging to the dominant party of the State.

---

*Deaths of Distinguished Medical Men.*—Thos. Southwood Smith, M. D., died on the 10th December last, at Florence, where he had been recently sojourning for the benefit of his health. The name of Soutwood Smith is familiar to the profession and the public largely, from his labors and success in sanitary reforms. He had reached the age of 73.

Sir John Cæsar Hawkins died November 9th, 1861, aged 89.

Dr. Cathcart Lee, of Dublin, December 16th, aged 50.

---

*Administration of Chloroform.*—Dr. Simpson, the introducer of chloroform as an anæsthetic, now recommends that a single layer of handkerchief be laid over the patient's face, and to let the chloroform fall on it drop by drop. It is claimed that there is less danger, a more speedy effect, and that a less quantity is required. Dr. Young stated that he kept a patient narcotized ten hours with two ounces and a-half.

---

*Application of Arsenic to Carious Teeth.*—G. W. Ellis, M. D., in the *Dental Cosmos*, states that arsenic is now the almost universally adopted medium for the destruction of the dental, and, in skilful hands, is thought to be divested of the many dangers which have been attributed to it. It appears that the most usual practice is to rub up the arsenic with kreosote and sulph. morphiæ. The value of the addition of morphia is much debated, but the weight of testimony is in its favor. The amount of arsenic employed varies from the twentieth to the fortieth of a grain, and the duration of the application varies from three to forty-eight hours, depending on the susceptibilities and peculiarities of each individual case. Dr.

Harris recommends it to be left in "about seven hours, or two hours after the tooth has completely ceased aching."

If, on the removal of the arsenic, the part be still sensitive, the renewal of the application is the practice mostly advocated.

---

*Local Anæsthesia.*—M. FOURNIER addresses the Academy of Sciences (Paris) on "Chloracetization." At the temperature of 63° Fah., he mixes equal parts of pure crystallizable acetic acid and chloroform, and by means of a wide-mouthed (or narrow, as the case requires,) applies it upon the surface where the epidermis is unbroken, and the phial being kept at the temperature of the hand, complete anæsthesia of the part will soon be induced. He suggests modifications of this process in cases where general anæsthesia is deemed inadmissible.

---

*Diagnosis of Parotitis and Phlegmon of the Face.*—M. TROUSSEAU recommends a method of distinction suggested by M. Archambault. Causing the patient to open the mouth widely so as to expose the mouth of Steno's duct by pressure of the parotid, a drop of pus can be expressed if the gland is the part affected. The process seems infallible in differential diagnosis.

---

*The Poison of Continued Fever.*—In a recent clinical lecture, Dr. Thomas K. Chambers remarked that he is "inclined to think that the usual path by which the poison enters, is the digestive canal. It is mixed with the saliva and carried down to the stomach, where it possibly may accumulate and be multiplied in the gastric mucus. During severe epidemics, it has been observed that those who smoke, that is, stop up their mouths with tobacco, and spit out the saliva instead of swallowing it, are less liable to be attacked. And in an early stage, even after the poison has begun to act upon the system, the fever may be arrested by emptying the stomach. Those who have watched my practice will have seen several instances of the success of this treatment, the fever cut short and convalescence entered upon immediately with its painless debility and emaciation gradually passing away."

Dr. Chambers, from careful analysis of urine in patients prior to and subsequent to the administration of an emetic, concludes that this remedial agency is capable of removing an essential part of the disease, even when the pathognomonic group of symptoms is present.

Before the emetic, the urine gave the usual evidences of destructive metamorphosis of tissue; after it the destruction is overcome by renewal. When this occurs so constantly as the result of a single dose, he finds it difficult to avoid the conclusion that it acts by removing from the mucous membrane a poison only partially absorbed and still adherent to it.

The strong resemblance between the incipient phenomena of continued fever, and those attending the ingestion of unwholesome material, is also cited. Again, the spontaneous vomiting, so very generally found in the first stage of the fever, seems to offer a presumption that the organ first impressed by the poison is the first to resent it.

The successful use of emetics which Dr. C. found to tally so exactly with his theory, unfortunately has not been realized by physicians generally, although emetics have long been held useful by them in the outset of continued fevers. We are inclined to the opinion that the effect of the substances given as emetics, has transcended the merely mechanical unloading of the stomach. Their generally relaxing and sedative character and the powerful influence they exert both through the sanguineous and nervous systems better explain to us their frequently efficient service. It seems more than likely that the emetic effect is only accidental and not necessary to the really valuable effect, any more than salivation is necessary to secure the alterant impression of mercury. However, upon this point there may be much said on both sides.

The effect of the poison received within the system is so graphically described, that we will present it.

"When the poison has once gained admission and is diffused by means of the circulation through the system, its effect is to destroy the vitality of a considerable amount of the organic living matter with which it comes in contact. The destruc·tion is interstitial, not local—I mean, it does not kill absolutely a certain spot which it touches, like sulphuric acid, but it kills only certain constituents of the tissues. The destruction is partial, not entire—the organic matter is not utterly disorganized, but only reduced to a less vital, less organic condition. It may be traced easiest in the alterations found in the medium by which it is diffused. The blood, the common thoroughfare for distribution of good and evil to the tissues, exhibits a serious change. If you examine it under the microscope, you will find that the normally shaped red disks are diminished in numbers as compared with what Pathologists call "melanosed" corpuscles, that is to say, dying or dead disks, shrivelled and small, of a dark color, with black specks in them, and with gimped edges. In bad cases these are unable to range themselves in rolls, as healthy blood does when it coagulates; they seem to have scarce any attraction for one another, and lie in amorphous heaps. They dissolve easily in the serum and form with it a red fluid. You may trace this dissolution in the dusky stain which the blood communicates to the skin in low fever.

"This poisoning goes on very gradually in some cases, and apparently quicker in others. You heard from this boy that he was five weeks ailing before he gave up work. There was an imperfect renewal of the body, evidenced in the languor after exertion and in the loss of appetite or deficient demand for new material by the formative processes. But destructive assimilation is not arrested, there is no stop to the removal of the effete tissues by excretion. I think it is possible that in a great many cases the disease, the partial death, may stop here, the destroyed tissues and their destroyer together be disorganized, be reduced to their elements and pass away. The idea is incapable of proof, but it would account for a vast number of those mysterious languors, unqualified, unnamed, and often unpitied, which distress patients and puzzle Doctors. However, when the poisoning has reacned a certain pitch, the nervous system cannot but take notice thereof, and express itself in the most common mode of taking notice of partial death, namely, by a shivering fit. Any severe injury to the body, a stretching of fibrous tissues, an operation, the fear of an operation, the absorption of destructive drugs, such as antimony, for example, will cause more or less of a rigor in proportion to the sensitiveness of the individual. And thus also when an interstitial death of some constituents of the body arrives at a certain point, there is a rigor. This rigor recurs from time to time at uncertain intervals, but generally about once a day.

"Then commences another symptom of partial death—pain. This boy described his head, his limbs, and his back as aching all at once. That is to say, wherever there was most tissue with sensitive nerves in it, there was pain, indicating the diseased state of that tissue. Now all this aching is a symptom of the earlier, rather than of the more advanced stages of fever; not because there is latterly less death, but because the nervous system becomes partially dead too, and does not feel so acutely.

"Observe that this patient tells us of nausea and loss of appetite, which diminished the food eaten—of vomiting, which rejected the greater part of that diminished food—and of diarrhœa, which carried off the remainder scarce digested at all. Yet in spite of this, the amount of solid matter passed from the kidneys is considerable; the specific gravity of the fifteen ounces of urine passed in the twenty-four hours is 1020. The metamorphosis, therefore, of the dead effete tissues into urea and salts is quite as active as in health. There is a continuous destruction of them in spite of the defective supply. This goes on as long as the fever poison lasts in the body, but when it is got rid of, the destruction ceases, no more is metamorphosed than is required to make room for new material, and the specific gravity of the urine falls during convalescence. This may take place very suddenly, as in the instance I gave you of a fever cut short by an emetic; but in general the alteration is more gradual.

"I have mentioned the large amount of urea in proportion to the nutrition in the urine of continued fever, which is rendered evident by its high specific gravity. There is also an increase very evident to the naked eye in another constituent of some importance, the colored organic material, which gives the secretion its ordinary hue. You saw how dark this boy's water was, and how deeply it stained the vessel from which I poured it on a piece of linen. There is great reason to suppose there is a close alliance between this substance and whatever gives the red tint to the blood-disks, and that its excess depends on excessive destruction of those important little living particles."

He observes that the sulphates and phosphates as also the chlorides of the urine are diminished.

The diarrhœa and exuded sanguineous serum drying upon the brown or livid tongue are further evidences of the interstitial decay and death. So also is the increased heat—the natural result of the chemical changes incident to the heightened rapidity of tissue metamorphosis.

The *materies morbi* is not the disease any more than the bullet which passes through the soldier's chest, or is extracted by the surgeon, is the disease which ensues. It is the partial death which has resulted which is the object of treatment. The physician is to enquire " What vitality is wanting, and where? What material is wanting, and where? And, How can I easiest supply them?"

The methods are obvious. First, remove the materies morbi so far as it may be reached. Second, supply moisture to the dry and heated surface. Third, supply nitrogenous food. Fourth, turn attention to the materia medica—and here, from theoretical considerations, but more especially as confirmed in value by practical experience, Dr. Chambers relies mainly upon free exhibition of dilute hydrochloric acid. He does not rank alcohol as part of the systematic *methodus medendi* of continued fever, but only as an adjuvant to the restorative treatment he advocates. " If there is very complete prostration, delirium of a low muttering character, it is required. A tremulous state of the muscles, marked especially by a quivering of the hands and figures, is a good test for the necessity of it; and so is a sharp, weak, unequal beat of the heart." Under these indications, the positive anæsthetic power of large doses of alcohol may be demanded.

Strangely enough, Dr. C. still adheres to the opinion that local bloodletting is advisable in the so called complications of typhoid.

The concluding words of this valuable lecture correspond so exactly with opinions we have long been in the habit of inculcating to students, that we quote them with unmixed pleasure:

"The moral of these cases is, do all you can to increase the appetite. Judge of the value of this drug and that drug, this tonic and that tonic, solely by the effect it has on the desire for food. If any remedy lessen this, insist upon leaving it off, whatever authorities may have recommended it; and form your judgment, not from tradition or prescription, but from its action in the individual case before you."

---

*Pertussis.*—Nitric acid is being revived as a remedy for whooping cough. Dr. H. Holmes, M. D. in a paper read before the Middlesex (Mass.) Med. Society (Bost. Med. Jour.) recommends the following: ℞ Tr. Cardamomi Comp. ℥ ss; Syrup. Simpl., ℥ iiiss., Acid. Nitrici, gtt. xxxij. M.; S. From five drops to one teaspoonful to be given frequently, according to the age of the patient, and the severity and frequency of the paroxysms.

---

*Evils of bad Drainage.*—It appears, from the investigations which have been made, that the cause of the lamented death of Prince Albert is traceable to the imperfect drainage and defective sanitary arrangements *in the neighborhood*, although not directly upon the site of the royal residence. Typhoid diseases are eminently the creatures of bad air, and in preventing this whatever precautions are taken must be not merely local but general and pervading. Not dependent on private caprice but public law.

---

*Commentaries on the Surgery of the War* in Portugal, Spain, France, and the Netherlands. From the battle of Rolica in 1808 to that of Waterloo in 1815; with additions relating to those in the Crimea in 1854 and 1855. Showing the improvements made during and since that period in the great art and science of surgery on all the subjects to which they relate. Revised to October 1855. By G. J. Guthrie, F. R. S. Sixth Edition. Philadelphia: J. B. Lippincott &

Co. 1862. Pp. 614 small octavo. For sale by S. C. Griggs & Co., Lake-st., Chicago.

It is safe to say that no other living man has had so wide a range of experience in military surgery as the author of this work. There is scarcely another living name so familiar to the ears of surgeons as his.

With the exception of Baron Larrey, there is perhaps no other name so intimately associated with military surgery as a speciality. And yet Surgeon Guthrie distinctily denies that "Surgery admits of any such distinctions." It is remarkable that Dr. Guthrie's second and extended course of lectures, on which the treatise before us is based, was distinctly recognized by the council of the Royal College of Surgeons as a course on General Surgery, and thus this book is particularly valuable as it contributes to general surgery the information derived by scientific observation of that particular class of casualties to which soldiers are exposed. He who is competent to be a civil surgeon is competent to be a military surgeon, and *vice versa.* The disciplined and scientific accountant can keep the books of a retail dry goods store or those of a government bureau, with equal facility after a few hours inspection. The same remark might be made with regard to diseases; the physician who is thoroughly competent to treat the diseases of one section, can full as easily treat those of another section; those of the camp as well as those of civil life.

We do not set out to praise or compliment this volume of Mr. Guthrie's, for we deem it unnecessary to "carry coals to Newcastle." We would only suggest that it is a book equally as valuable to the surgeon in civil life as to the military surgeon, and we know of no other surgical work which, in as limited space, conveys so vast an amount of inestimable information. It is not a catch-penny thrown upon the war market, but one which will be ranked with the text books and classics of professional literature.

*Excessive Lochial Discharge.*—"Well, then, you have a feeble, anæmic patient, and the lochial discharge continues profuse and of bright color, six or eight days after labor. You find the uterus remaining above the pubes, nearly as large as a child's head. You give her ergot, as I before described, and, in addition, you make a prescription something like the following: ℞, Quiniæ Sulph. ʒj; Ferri Sulph. gr. xij; Ext. Nucis Vomic., Pulv. Capsici aa gr. vj. M. Ft. pil (argent) No. 12. S. One three times a day, directly after eating. Now ask yourselves what is the object of the quinine, the iron, the nux vomica, the capsicum? I have seen, quite frequently, this condition associated with a very profuse lactation, which is an additional drain upon the system, and the patient is nervous, irritable, and suffers from headache and insomnia. Now, what advantage will you obtain by adding to the above formula four grains of opium?"

Thus says Prof. B. Fordyce Barker, in a recent clinical lecture; and beyond the useful prescription contained, the paragraph suggests an idea worthy of assimilation. Medicinal formulas are very convenient for routine practitioners, but their use endangers the overlooking of the peculiar points of individual cases.

Unfortunately, notwithstanding the deserved ridicule which has been thrown upon excessively complex medical combinations, examination of any apothecary's book will show that polypharmacy is still a prevailing vice. Some of it springs from imitation of English apothecary practice, where the practitioner, not having a legal claim for his advice, finds it necessary to secure his honorarium through a diversity of prescriptions.

Some of it finds its origin in a weak affectation of wisdom, which is supposed to be at its culminating point in a recipe, multitudinous in composition, and appropriately contracted in professional Latin. We have known many such heterogenous conglomerations, pointed out by the admiring patrons of an aspiring physician, as the most thoroughly incontrovertible evidence of his exceeding wisdom. Some of it has descended from the fathers, and we hesitate to banish it, as we hesitate to burn the worthless heirlooms which fill our old garrets.

Some of it arises from the uncertainty existing in the practitioner's own mind about what is best to do, and he means *to hit somewhere.*

Some of it finds birth in consultations—the creature of compromise. Who has not seen it?

Some of it comes from—but we forbear as the subject grows upon us. Meanwhile we commend Prof. Barker's hint to general consideration.

---

*A Medico-Legal Point.*—Prof. Alonzo Clark, commenting on a recent case of suspected homicide, states that it is "safe to infer that neither blood nor bloody effusion in the pleuritic cavities is ever the direct result of suspended respiration, no matter how produced; that when such effusion is found, except when attended by laceration of the lung through violence, it is always either a pathological condition and attended by particular symptoms during life, or a post mortem result that may be considered in the light of a drainage. Even in drowned persons, in whom it is most frequently met with, there is no evidence that it is ever observed till after days, and many times weeks, have elapsed."

This is a point of considerable importance, and may be considered as thoroughly settled. Nevertheless, we have known several cases in which this bloody effusion in the pleural cavity was thought conclusive evidence of infanticide. The person may have been murdered, but this circumstance is evidence only of ante-mortem disease or post-mortem decomposition.

